# Experimental and Theoretical Study on Theobromine Solubility Enhancement in Binary Aqueous Solutions and Ternary Designed Solvents

**DOI:** 10.3390/pharmaceutics13081118

**Published:** 2021-07-22

**Authors:** Tomasz Jeliński, Dawid Stasiak, Tomasz Kosmalski, Piotr Cysewski

**Affiliations:** 1 Department of Physical Chemistry, Pharmacy Faculty, Collegium Medicum of Bydgoszcz, Nicolaus Copernicus University in Toruń, Kurpińskiego 5, 85-950 Bydgoszcz, Poland; 288355@stud.umk.pl (D.S.); Piotr.Cysewski@cm.umk.pl (P.C.); 2 Department of Organic Chemistry, Pharmacy Faculty, Collegium Medicum of Bydgoszcz, Nicolaus Copernicus University in Toruń, A. Jurasza 2, 85-089 Bydgoszcz, Poland; tkosm@cm.umk.pl

**Keywords:** theobromine, solubility, COSMO-RS, NADES, methylxanthines

## Abstract

The solubility of theobromine was studied both experimentally and theoretically. The solubility was determined spectrophotometrically at 25 °C in neat organic solvents, aqueous binary mixtures, Natural Deep Eutectic Solvents (NADES) and ternary NADES mixtures with water. It was found that addition of water in unimolar proportions with some organic solvents increases theobromine solubility compared to neat solvents. Additionally, using NADES results in a solubility increase of the studied compound not only in relation to water but also DMSO. The addition of water (0.2 molar fraction) to NADES is responsible for an even larger increase of solubility. The measured solubilities were interpreted in terms of three theoretical frameworks. The first one—belonging to the set of data reduction techniques—proved to be very efficient in quantitative back-computations of excess solubility of theobromine in all studied systems. The default approach utilizing the well-recognized COSMO-RS (Conductor-like Screening Model for Real Solvents) framework offered at most a qualitative solubility description. The extended search for possible contacts provided evidence for the existence of many intermolecular complexes that alter the electron density of the solute molecule, thus influencing solubility computations. Taking into account such intermolecular contacts by using the COSMO-RS-DARE (Conductor-like Screening Model for Realistic Solvation-Dimerization, Aggregation, and Reaction Extension) framework seriously increased the accuracy of solubility computations.

## 1. Introduction

**Figure 1:**
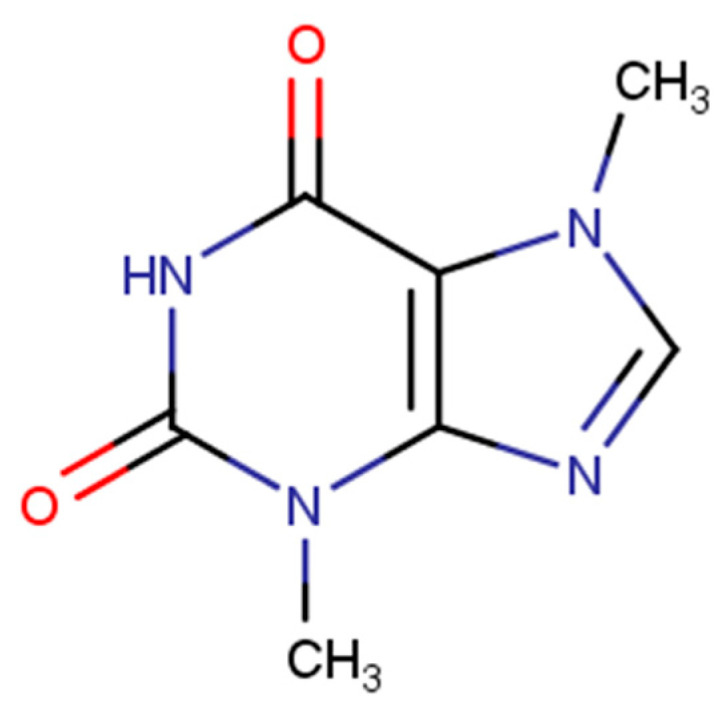
Structural formula of theobromine.

Methylxanthines can be classified as purine alkaloids, having a purine moiety, i.e., a fused heterocyclic system comprising annulated pyrimidine and imidazole rings, as their molecular core. The compound discussed in this particular study, i.e., theobromine, is a disubstituted derivative of xanthine (3,7-dimethylxanthine, MW = 180.167 g∙mol^−1^, structural formula in Figure 1). Methylxanthines belong to a group of compounds that are widespread in nature, particularly residing in tea and other plant leaves, coffee and cocoa beans, as well as cola seeds [1]. Theobromine in particular can be found mostly in cocoa and chocolate [2]. It was discovered in 1841 but its synthesis was achieved as late as 1882. Methylxanthines play a crucial role in several biological processes with the stimulation of the central nervous system, increase of blood pressure, diuresis of kidneys, relaxation of smooth muscles, strengthening of the concentration of skeletal muscles or gastric acid secretion [3,4] being their most important biological activities. The mode of action of methylxanthines arises from their activities as phosphodiesterase inhibitors [5] and nonselective adenosine receptor antagonists [6]. While theobromine is currently not used as a prescription drug [7], it attracts the attention of researchers [8,9,10,11,12,13] since it possesses several beneficial properties related to human health, such as antioxidant [14], anti-inflammatory [15] or immunomodulatory [16] activities. A very important and widely explored property of theobromine is its anti-cancer activity [17,18,19,20]. Theobromine may occur in humans as a result of caffeine metabolism in the liver where itself is metabolized into xanthine [2]. Theobromine has limited solubility in water (330 mg/L at 25 °C [21]), a low octanol–water partition coefficient (logP = −0.78 [22]) and is an amphoteric compound [1]. It also has a significantly higher melting point than caffeine and theophylline (M_p_ = 357 °C [23]).

One of the main challenges in many areas of modern industry, including pharmaceutics, is the problem of limited solubility of different chemical compounds, especially in aqueous media but also in other solvents. As many substances on the market are classified as insoluble in aqueous solutions [24,25,26], it is necessary to develop techniques overcoming this limitation. Many approaches have been proposed to address this challenge, including micronization [27], monocrystal formation [28,29], amorphization [30,31,32], complexation with cyclodextrins [33,34], solid dispersion formation [35], pH modification [36,37], formation of salts [38,39] and cocrystals [40,41,42,43]. A very promising and simple way of increasing solubility is the usage of cosolvation techniques [44,45]. The cosolvation effect is achieved when an addition of some amount of a particular solvent to the primary solvent results in increased solubility of the solute [46]. Different organic compounds can be used as cosolvents, including alcohols, propylene glycol or glycerin [47,48]. There are however limitations of using organic solvents, partially because of their potential toxicity. This results in the need for different solvents that would have a satisfactory solubilizing potential and at the same time possess desirable properties related to environmental and health safety. Natural Deep Eutectic Solvents (NADES) fulfill these dual requirements and have focused the attention of researches for some time. In general, Deep Eutectic Solvents (DES) have a lower melting point than their individual components, which allows them to remain in liquid state even at room temperature [49]. In particular, natural deep eutectic solvents can be defined as bio-based DES with their constituents belonging to such classes of compounds as organic acids, alcohols, amino acids, sugars or other plant based primary metabolites [50,51]. Thanks to their desirable physicochemical properties, such as low volatility, sustainability and biodegradability, low cost and simplicity of preparation and the potential of fine-tuning for specific applications, NADES are very attractive from the point of view of efficiency, safety and economics [52,53,54,55]. It is then no surprise that NADES have found their place in many different applications [54,56,57,58,59,60,61].

The theoretical studies of solubility are very important both in the context of predicting the actual solubility in a variety of systems, as well as gaining insight into the phenomena occurring during these processes. The number of different solubility models is extensive and encompasses many different approaches to solubility predictions [62,63,64,65,66,67,68,69,70,71]. Among the most widely utilized approaches is the COSMO-RS model [72,73]. This approach was used for the prediction of solubility [74,75], solvent screening [76,77,78], as well as describing multiphase chemical equilibria [79,80,81]. It relies on the combination of the density functional theory calculations and statistical thermodynamics as post treatment [82], becoming a standard approach in many scientific and industrial applications.

The aim of the study was threefold. First of all, the solubility of theobromine in a variety of solvents was determined, including neat organic solvents, aqueous binary mixtures, natural deep eutectic solvents and NADES mixtures with water. Secondly, excess solubility modeling was applied to the collected dataset and different approaches were compared. Finally, the COSMO-RS-DARE approach, which relies on the direct inclusion of pairs representing the most stable structures at saturated conditions, was utilized for predicting solubility and its performance was assessed. The obtained results, both experimental and computational, while related to theobromine solubility in particular, will also have a general character pointing out to the possible routes for solubility improvement of different compounds and important considerations regarding theoretical solubility predictions using the COSMO-RS framework.

## 2. Materials and Methods

### 2.1. Materials

Theobromine (CAS: 83-67-0) used as a solute in this study was purchased from Sigma-Aldrich (Darmstadt, Germany) with a purity of ≥98%. Several organic solvents were used in the study, namely dimethyl sulfoxide-DMSO (CAS: 67-68-5), dimethylformamide-DMF (CAS: 68-12-2), 1,4-dioxane (CAS: 123-91-1), acetonitrile (CAS: 75-05-8), acetone (CAS 67-64-1), methanol (CAS: 67-56-1), 1-propanol (CAS: 71-23-8), 1-butanol (CAS: 71-36-3), 1-pentanol (CAS: 71-41-0) and ethyl acetate (CAS: 141-78-6). All of the solvents were supplied by Avantor Performance Materials (Gliwice, Poland), Poland with a purity of ≥99% and were used without any initial procedures. The following chemicals were supplied by Sigma-Aldrich and used in preparation of Natural Deep Eutectic Solvents: choline chloride (CAS: 67-48-1), glucose (CAS: 50-99-7), fructose (CAS: 57-48-7), sorbitol (CAS: 50-70-4), xylitol (CAS: 87-99-0), maltose (CAS: 69-79-5), saccharose (CAS: 57-50-1), glycerol (CAS: 56-81-5). All of NADES constituents had a purity of ≥99%.

### 2.2. Preparation of Calibration Curve

In the initial step, a stock solution of theobromine in methanol was prepared at a concentration equal 1.6 mg/mL in a 100 mL volumetric flask. Next, predefined volumes of this solution were successively diluted in 10 mL volumetric flasks using methanol and in this way several solutions of theobromine with decreasing concentration were obtained. These solutions were measured spectrophotometrically and a relationship between theobromine concentration and the values of absorbance measured at a wavelength of 272 nm was obtained. Three distinct curves were prepared, and their average was used as the calibration curve utilized for determining the concentration of theobromine in the studied samples. Additionally, statistical parameters such as the determination coefficient R^2^, limit of detection (LOD) and limit of quantification (LOQ) were calculated. The detailed values of concentration and absorbance, as well as the obtained parameters of the curve can be found in Table S1a,b, respectively in Supplementary Materials.

### 2.3. Preparation of Samples in Organic Solvents and Their Mixtures with Water

The preparation of solvent mixtures involved mixing preset amounts of both organic solvent and water in 10 mL volumetric flasks in different molar ratios. Next, an excess amount of theobromine was placed in test tubes in order to obtain saturated solutions. These test tubes were then filled either with a neat solvent or a solvent mixture prepared as described above. For each tested system, three samples were prepared. The samples were incubated for 24 h in an Orbital Shaker Incubator ES-20/60 supplied by Biosan at 25 °C. The temperature was adjusted with an accuracy of 0.1 °C and the temperature variance in a daily cycle was ±0.5 °C. The samples were simultaneously mixed at 60 rev/min in the incubator. After incubation, filtration of the samples took place using a PTFE syringe filter with 0.22 μm pore diameter. In order to sustain a temperature similar to the one of the samples, all of the test tubes, syringes, filters, etc. were pre-heated in the same conditions. A fixed amount of the obtained filtrate was transferred to test tubes containing methanol and samples diluted in such way were than measured spectrophotometrically. For the determination of the molar fractions of Theobromine also the density of the samples was measured.

### 2.4. Preparation of Samples in NADES and Their Mixtures with Water

The preparation of Natural Deep Eutectic Solvents (NADES) involved mixing choline chloride and the second constituent in different molar ratios. The obtained mixtures were transferred to sealed test tubes and placed in a water bath with a temperature of 90 °C until the formation of a uniform solution. Again, an excess amount of theobromine was added to the test tubes containing both pure NADES, as well as their mixtures with water, in which the NADES was treated as a cosolvent and added in varying molar ratios. The samples were then incubated for 24 h at 25 °C, similarly as for organic solvents. The increased viscosity and density of NADES required the samples to be centrifuged at 1000 rev/min for 5 min with the use of EBA 20 centrifuge from Hettich for the undissolved precipitate to remain on the bottom of the test tube. Further procedure included filtration and spectrophotometric measurements, as it was the case for organic solvents.

### 2.5. Solubility Measurements

The concentration of theobromine in the samples was determined via a spectrophotometric method using a A360 spectrophotometer from AOE Instruments. The spectra were recorded at a wavelength range from 190 to 700 nm with a 1 nm resolution. The spectrophotometer was calibrated using methanol before each measurement. The samples were diluted with methanol in order to achieve absorbance values within the linearity limit of the method. Based on the linear equation obtained for the calibration curve and the absorbance values measured at 272 nm it was possible to determine the concentration of Theobromine in the samples. For each system three samples were measured and their mean concentrations, expressed as mole fractions, were determined along with standard deviation values.

### 2.6. FTIR-ATR Measurements

Solid samples of theobromine were analyzed by FTIR-ATR measurements after collecting sediments from the test tubes after the solubility experiments described above. The samples were dried and measured using a FTIR Spectrum Two spectrophotometer from Perkin Elmer, which was equipped with a diamond attenuated total reflection (ATR) device. The spectra were recorded in 450–4000 cm^−1^ wavenumber range.

### 2.7. COSMO-RS Solubility Computations

The COSMO-RS (Conductor-like Screening Model for Real Solvents) framework belongs to QM continuum solvation models that are augmented with statistical thermodynamics analysis of electrostatic surface interactions. It enables direct calculations of chemical potentials of components in the bulk phase. Due to the widespread popularity of this approach, it is not necessary to provide its fundamentals as they can be found in original publications [73,83,84]. However, it is crucial to state that the microscopic properties obtained with an aid of the quantum chemical part of this approach are directly connected to different thermodynamic macroscopic properties of the liquid by means of statistical thermodynamics through analyzing the densities probability distributions, which are termed σ-profiles and σ-potentials. By integrating the latter over the surface one obtains a combinatorial contribution to the chemical potential, which allows to predict many different thermodynamic properties, such as activity coefficients, excess properties or partition coefficients, as well as solubility. In order to conduct actual calculations of the mentioned properties, a proper representation of the molecular structure is required. Usually, it is conducted by a thorough exploration of the conformational space using COMSOconf software, which generates the most energetically favourable structures of a specific molecule. Here, the computations were conducted using TURBOMOLE rev. V7.5.1 interfaced with BIOVIA TmoleX 2021 (version 21.0.1) (Dassault Systèmes, Biovia, San Diego, CA, USA) using RI-DFT BP86 (B88-VWN-P86) functional and def-TZVP basis set for geometry optimization and def2-TZVPD basis set for single point calculations with inclusion of fine grid marching tetrahedron cavity 90. Additionally, the parameter sets with hydrogen bond interaction were included and van der Waals dispersion terms were quantified using the “D3” method of Grimme et al. [85]. All of the calculations of solubility were performed using COSMOtherm (version 20.0.0, revision 5273M) [86] with BP_TZVPD_FINE_20.ctd parametrization.

Solubility computations of solid solutes were performed by iteratively solving the equation below:

(1) $$\log\left(x_{i}^{s,\left(1\right)}\right)=\frac{\mu_{i}^{p}-\mu_{i}^{s}\left(x_{i}^{s,\left(0\right)}\right)-\max\left(0,\Delta G_{fus}\right)}{RTln\left(10\right)}$$

where *μ_i_^o^* stands for the chemical potential of a pure compound *i*, *μ_i_^s^*, (*i*) is the solute chemical potential generated after i-th iteration, while Δ*G_fus_* represents the solute Gibbs free energy of fusion. The zeroth order solution of Equation (1) corresponds to solubility at infinite dilution *x_i_^s^^,∞^* ≅ 1/γ∞ and is valid only for small concentrations of the solute. For improving accuracy, the obtained solubility is used for a re-definition of the chemical potential and substitution into Equation (1) until self-consistency is achieved. It is clear that the knowledge of the exact or at least approximated value of fusion Gibbs free energy must come from outside of the COSMO-RS method since it characterises the properties of bulk liquid phases.

### 2.8. COSMO-RS-DARE Computations

Applying the COSMO-RS framework for prediction of properties of multi-component systems can be hampered by the difficulty arising from the possibility of direct intermolecular interactions leading eventually to the formation of new complexes between solute molecules, as well as solute and solvent molecules. Although the default definition of the input files does not usually contain these species, they can be important because the mentioned complex formation may affect the charge densities. This problems is addressed by COSMO-RS with the Dimerization, Aggregation, and Reaction Extension acronymed as COSMO-RS-DARE [87]. This approach makes it possible to extend the conformers list for a specific compound by including the products of any reaction in which the σ-surface of only one molecule is included and the other one is weighted out. This can be obtained by definitions of so called “mcos” files, which have atomic weights set to one for atoms of the species in question and to zero for atoms of the second component of interactions. Therefore, the number of atoms in such pseudo-conformers is the same as it was in the monomers. It is also important to point out that the default settings of COSMO-RS do not capture the interactions between σ-surface segments of these pseudo-conformers and a new descriptor is required in order to ensure that all new surface segments are included in the computations. The above is conducted by the introduction of two adjustable parameters in the formal form of the Gibbs free energy interaction between i-th and j-th compounds:

(2) $$\Delta G_{ij}^{int}=H_{ij}^{int}-TS_{ij}^{int}$$

Hence, the methodology offered by COSMO-RS-DARE relies on replacing the common interaction energy between surface segments, which includes misfit, hydrogen bonding and van der Waals forces, with the above formula related to the interaction of two dimerization segments. The parameter values in Equation (2) can be estimated through the fitting of the computed solubility values to the experimental ones for each considered solvent and temperature. As the usage of COSMO-RS-DARE protocol for solubility computations was already successfully tested [88], the methodology was extended in this study to also include binary solvents mixtures. This procedure relies on treating the considered solvent compositions as individual solvents. Additionally, for the performed COSMO-RS-DARE computations only the pairs formed by theobromine itself or with solvent molecules were taken into account, as well as only one interaction parameter was set for all such pseudo-conformers. The first of these simplifications ignores contacts resulting from the interactions of solvent molecules while the second one assumes that the electron density changes upon formation of complexes are of similar magnitude.

Obviously, obtaining the “mcos” files requires performing a conformational screening of bimolecular systems. Such bimolecular clusters were obtained through the generation of the most probable structures of their complexes by applying the criterion of the highest probabilities of contacts between the two molecules. The computations of contact statistics based on probability of interactions between the surface segments of the molecule, as offered by the COSMOtherm program, are invoked with the CONTACT = {1 2} ssc_probability ssc_weak ssc_ang = 15.0 command. Hence, the dihedral angle value between the two contacting molecules in the cluster varies with a 15° step and the geometries resulting from these calculations were stored for additional geometry optimization and clustering. For a typical conformational analysis conducted for monomers the same methodology was adopted.

### 2.9. Statistical Measures

Two statistical parameters were used as the criteria for assessing the accuracy of the utilized solubility prediction approaches. The first one was the root-mean-square deviation (RMSD), defined as follows:

(3) $$RMSD=\sqrt[{2}]{\frac{1}{N}\sum_{i=1}^{N}{\left(x_{i}^{est}-x_{i}^{exp}\right)}^{2}}$$

where *x_i_^est^* is the estimated mole fraction value, *x_i_^exp^* is the experimental mole fraction value and *N* is the number of experimental points. The other parameter was the mean absolute percentage error (MAPE), which is defined by the following formula (adopting the same notation):

(4) $$\;MAPE=\frac{1}{N}\sum_{i=1}^{N}\left|\frac{\left(x_{i}^{est}-x_{i}^{exp}\right)}{x_{i}^{exp}}\right|\cdot 100$$

## 3. Results and Discussion

### 3.1. Solid State Characteristics

**Figure 2:**
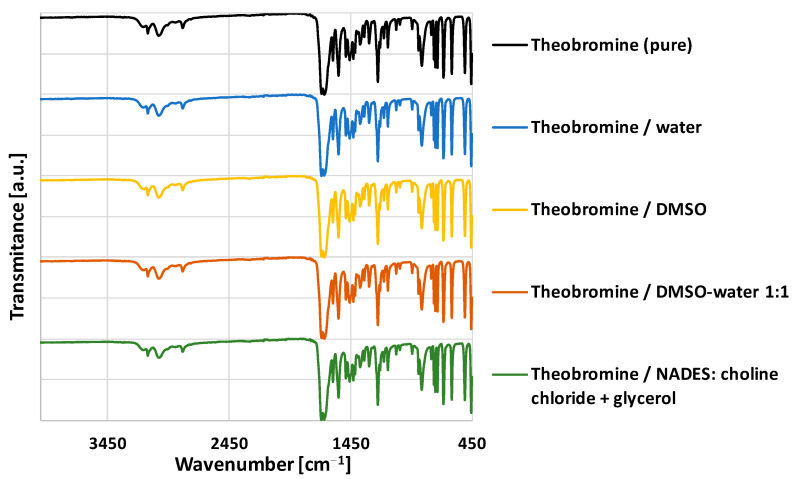
FTIR-ATR spectra of theobromine sediments obtained in selected solvents.

Theobromine exists in only one stable crystal form [89] and crystallizes in P21/c system with a = 9.2990, b = 18.6980, c = 9.0381 Å and with Z = 8 [90]. No polymorphism was determined for this compound. Additionally, contrary to caffeine [91] and theophylline [92], it does not easily form hydrates and remains in its anhydrous form even immersed in water for 24 h [93]. It is then expected that no changes in the form of theobromine will occur when comparing the pure compound initially used for solubility studies and the sediment obtained after experiments. However, in order to confirm this, a series of FTIR-ATR experiments were performed and selected results were summarized in Figure 2, which shows the FTIR spectra of Theobromine residues after measuring its solubility in different solvents.

If some new molecular complexes in the solid phase, such as solvates, are formed shifts of the absorption bands are expected, compared to the pure compound. As it can be seen in Figure 2, the FTIR spectra of solid residues obtained after solubility measurements are in agreement with the spectrum of the pure compound with no significant deviations in the position of the bands, as well as without any new bands. The characteristic IR absorption bands for theobromine can be attributed to N–H stretching of the pyrimidine ring (3150 and 3114 cm^−1^), carbonyl groups C=O stretching (1660 and 1557 cm^−1^) and C=N stretching (1687 cm^−1^) vibrations. Based on the above, it can be stated that theobromine remains in its single stable crystal form during the experiments without the formation of solvates or hydrates.

### 3.2. Experimetnal Solubility of Theobromine

**Figure 3:**
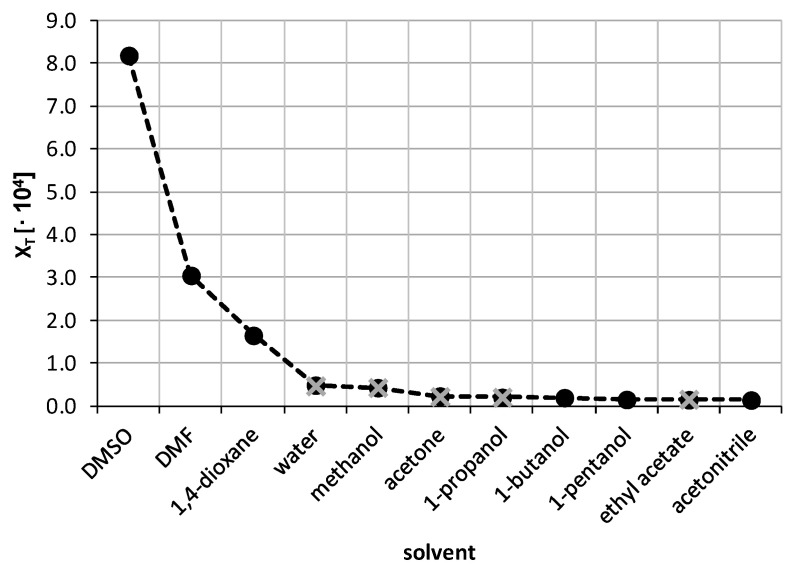
Solubility of theobromine at 25 °C, expressed as its mole fraction, in ten neat organic solvents and water. Black circles represent measurements of this work, while gray crosses indicate values published by Zhong et al. [94].

The first stage of solubility measurements of theobromine encompassed the usage of ten neat organic solvents and water. The obtained solubility values of theobromine are presented in Figure 3. In order to confirm the reliability of the proposed methodology it was necessary to conduct a comparison with available literature data. Several values of theobromine solubility in different solvents taken from [94] were compared to results obtained in this study. The results seem to be in good accordance with each other, since the mean relative difference between the two datasets is only 2.60%. For detailed results please refer to Table S2 in Supplementary Materials.

The highest solubility of theobromine was found in the case of DMSO, namely x_T_ = (8.20 ± 0.04)·10^−4^; c = 2.09 ± 0.02 mg/mL. Additionally, using DMF and 1,4-dioxane resulted in solubility values greater than the one for water, namely x_T_ = (3.05 ± 0.05)·10^−4^; c = 0.72 ± 0.01 mg/mL and x_T_ = (1.65 ± 0.03)·10^−4^; c = 0.49 ± 0.01 mg/mL, respectively. Interestingly, water was responsible for the fourth largest Theobromine solubility, ahead of the other seven organic solvents, with x_T_ = (0.49 ± 0.01)·10^−4^; c = 0.48 ± 0.01 mg/mL. The three most effective organic solvents were selected for further studies during which they formed binary solvents with water with varying proportions. Additionally, two other solvents were selected for this task, i.e. methanol (x_T_ = (0.43 ± 0.01)·10^−4^; c = 0.19 ± 0.01 mg/mL) and acetone (x_T_ = (0.23 ± 0.01)·10^−4^; c = 0.06 ± 0.01 mg/mL).

**Figure 4:**
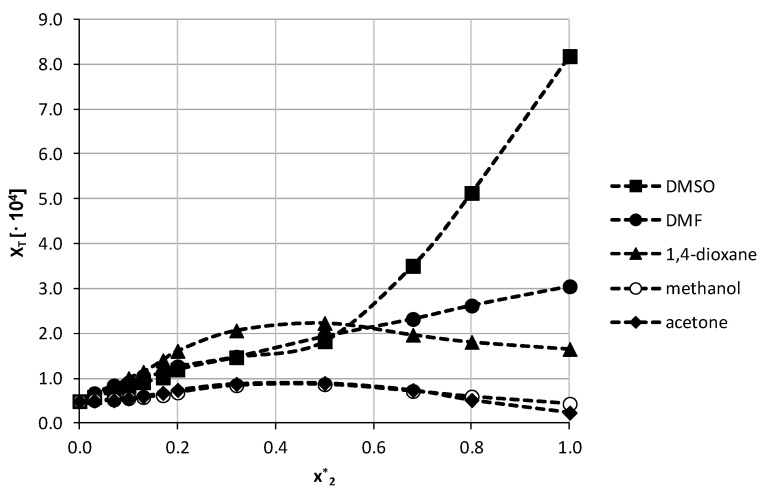
Solubility of theobromine at 25 °C, expressed as its mole fraction, in binary solvents comprising water and five different organic solvents in varying compositions. On the abscissa, $x_{2}^{*}$ represents the mole fractions of organic solvent in solute free binary solutions.

The aforementioned five organic solvents were used to create binary solvents with different molar ratios of water and the solvent. A total of twelve data points was selected, including the neat solvent and water. The corresponding results are collected in Figure 4.

When analyzing the obtained results, it is evident that two different patterns are observed. For DMSO and DMF no cosolvation effect was obtained as the highest solubility was found for the neat solvent (x_T_ = (8.20 ± 0.04)∙10^−4^; c = 2.09 ± 0.02 mg/mL and x_T_ = (3.05 ± 0.05)∙10^−4^; c = 0.72 ± 0.01 mg/mL, respectively). There can be observed a gradual increase of theobromine solubility with increasing organic solvent content, although for DMF it is almost a linear increase, while in the case of DMSO two linear sections are visible, with an inflection point at the organic solvent content of $x_{2}^{*}\;$= 0.5. A different behavior was found for the three remaining organic solvents and in these cases a solubility maximum was achieved for a water-solvent composition with a molar ratio equal 1:1. For 1,4-dioxane the solubility of theobromine in the most optimal composition was found to be x_T_ = (2.23 ± 0.01)·10^−4^; c = 0.92 ± 0.01 mg/mL, while for acetone and methanol it was x_T_ = (0.89 ± 0.03)·10^−4^; c = 0.35 ± 0.01 mg/mL and x_T_ = (0.87 ± 0.02)·10^−4^; c = 0.53 ± 0.01 mg/mL, respectively. Interestingly, for the last two solvents the increasing solubility order is different than for neat solvents. Additionally, the general trend of solubility changes is different for 1,4-dioxane than for the two latter solvents. After reaching the point of maximum solubility, the values of theobromine mole fraction in the mixture decrease only slightly in the case of 1,4-dioxane, and the value of solubility in the neat solvent is equal almost 74% of the maximum solubility value achieved at $x_{2}^{*}\;$= 0.5. For methanol and acetone a much more pronounced decrease of solubility is observed and the solubilities for neat solvents are 50% and 26% of the maximum value, respectively. Detailed results of theobromine solubility in both neat organic solvents and binary solvents with water are presented in Tables S3 and S4 in Supplementary Materials.

**Figure 5:**
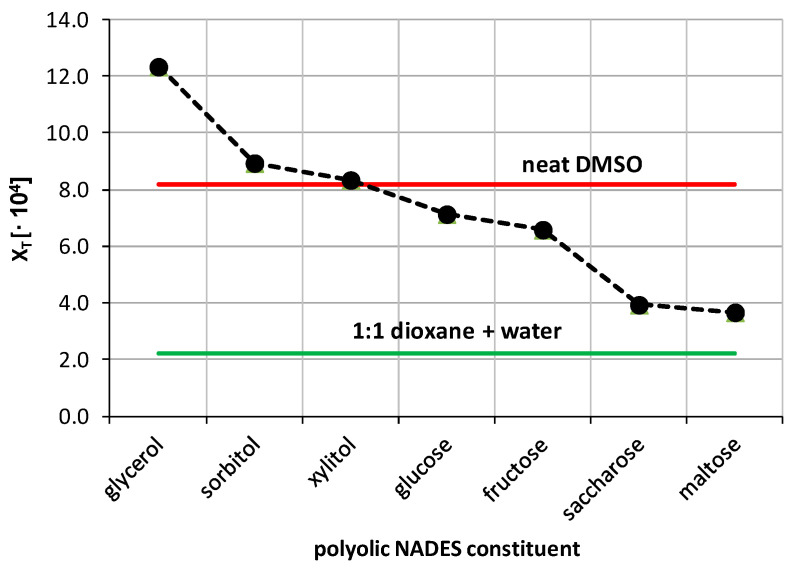
Mole fraction solubility of theobromine determined in water-free natural deep eutectic solvents at 25 °C. All designed solvents were prepared in unimolar proportions. For comparison, the solubilities in neat DMSO and 1:1 dioxane-water mixture were highlighted.

The solubility of theobromine was studied not only in organic solvents but also in natural deep eutectic solvents and their mixtures with water. In the initial phase of this part, the solubility of theobromine was measured in NADES comprising choline chloride and one of seven other constituents, belonging to the class of polyols, in a molar ratio equal 1:1. The obtained results were collected in Figure 5.

The highest solubility of theobromine was found for the NADES comprising choline chloride and glycerol, i.e., x_T_ = (12.34 ± 0.68)·10^−4^; c = 3.37 ± 0.06 mg/mL. Lower and similar results were obtained when using sorbitol and xylitol as the second NADES constituent and in these cases x_T_ = (8.94 ± 1.20)·10^−4^; c = 2.14 ± 0.03 mg/mL and x_T_ = (8.34 ± 0.94)·10^−4^; c = 2.09 ± 0.03 mg/mL, respectively. Two other NADES with similar solubility values included the ones with glucose and fructose, resulting in theobromine solubility equal x_T_ = (7.14 ± 0.76)·10^−4^; c = 1.65 ± 0.02 mg/mL and x_T_ = (6.59 ± 0.72)·10^−4^; c = 1.52 ± 0.01 mg/mL, respectively. The first three natural deep eutectic solvents resulted in theobromine solubility greater than in the case of DMSO, which was characterized with the largest solubility among the studied organic solvents. Additionally, all of the studied formulations were characterized by greater solubility of theobromine than the 1:1 dioxane-water mixture. The first four most effective NADES compositions were chosen for further studies, namely their usage in mixtures with water, as it was done previously with organic solvents.

**Figure 6:**
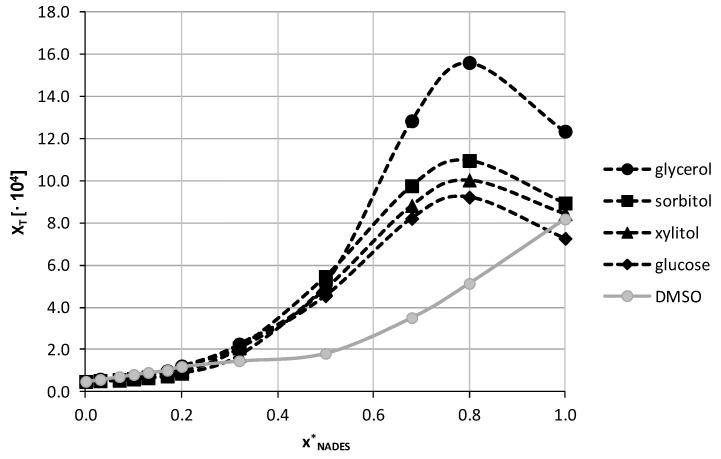
Solubility profile of theobromine at 25 °C in mixtures comprising NADES successfully diluted with water. On the abscissa, $x_{\mathrm{NADES}}^{*}$, represents values of the mole fractions of natural deep eutectic solvent in aqueous solutions. For comparison purposes solubility in DMSO + water was plotted as a function of $x_{2}^{*}$ (gray line).

In Figure 6 there were collected the results of theobromine solubility in mixtures containing water and natural deep eutectic solvents made of choline chloride and one of the selected four polyols in unimolar proportions.

For all of the studied NADES the largest solubility of theobromine was found for the water-NADES composition with $x_{\mathrm{NADES}}^{*}$= 0.8. In this point the order of increasing solubility is the same as for pure NADES, namely glucose > xylitol > sorbitol > glycerol and the differences are most pronounced for the compositions yielding the largest solubilities. For glycerol the largest solubility was equal x_T_ = (15.59 ± 0.17)·10^−4^; c = 5.12 ± 0.07 mg/ml, for sorbitol x_T_ = (10.96 ± 0.03)·10^−4^; c = 3.06 ± 0.01 mg/ml, for xylitol x_T_ = (10.03 ± 0.05)·10^−4^; c = 2.99 ± 0.02 mg/ml and for glucose x_T_ = (9.22 ± 0.08)·10^−4^; c = 2.56 ± 0.02 mg/ml. For the most efficient composition, including choline chloride and glycerol, the obtained solubility of theobromine was almost 32 times larger than for water and about 1.5 times larger than for neat DMSO. The pattern of solubility changes is different for NADES comprising glycerol than for the other three with a more pronounced solubility increase starting from $x_{\mathrm{NADES}}^{*}\;$= 0.8. In all studied cases also the composition with NADES content equal $x_{\mathrm{NADES}\;}^{*}$= 0.68 resulted in larger solubilities than pure natural deep eutectic solvents, although the difference was quite small. Detailed results of theobromine solubility in both pure NADES and NADES-water mixtures can be found in Tables S5 and S6 in Supplementary Materials.

### 3.3. Excess Solubility Modeling

**Figure 7:**
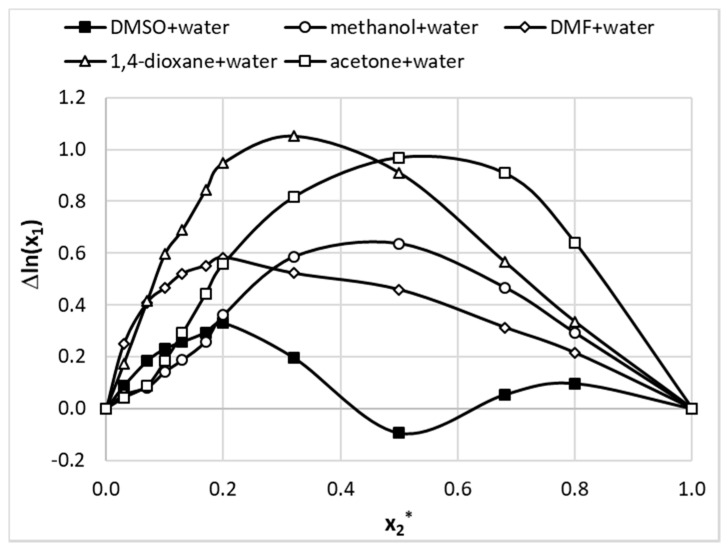
Excess solubility values of theobromine ($x_{1}$) dissolved in aqueous binary mixtures at room temperature. On abscissa, $x_{2}^{*}$ denotes solute free mole fraction of the organic solvent.

**Figure 8:**
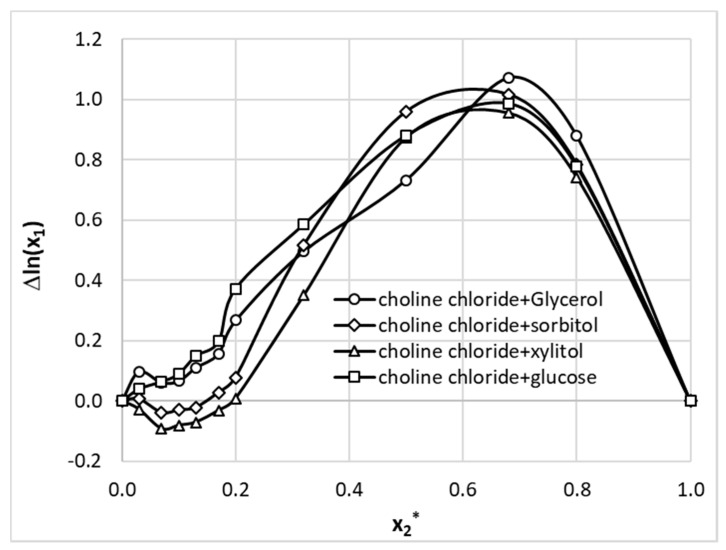
Excess solubility values of theobromine ($x_{1}$) dissolved in aqueous ternary mixtures formed at room temperature. On abscissa, $x_{2}^{*}$ denotes solute free mole fraction of the 1:1 NADES in aqueous formulations.

Solubility interpretation both in binary and ternary solvent mixtures was performed using excess solubility terms. This approach enables for quantification of mutual solvents interference and accounting for the non-additivity effects. The positive values indicate that the cooperative effect of solvents mixture promotes solubility compared to neat solvents. To the contrary, negative values suggest anti-cooperativity of solvents. The amount of this effect can be inferred from deviations from the algebraic rule of mixing according to the following formula:

(5) $$ln\left(x_{1}^{bin}\left(T\right)\right)=\left(1-x_{2}^{*}\right)\cdot ln\left(x_{1}^{neat}\left(T,x_{2}^{*}=0\right)\right)+x_{2}^{*}\cdot ln\left(x_{1}^{neat}\left(T,x_{2}^{*}=1\right)\right)+\Delta^{exc}ln\left(x_{1}^{bin}\left(T\right)\right)$$

where $x_{2}^{*}$ represents the mole fraction of the organic solvent in aqueous mixture. The last term in the above equation,$\;\Delta^{exc}ln\left(x_{1}^{bin}\left(T\right)\right)$, accounts for the non-additivity of solubility in the binary mixture with respect of neat solvents. As the ratio of choline chloride and polyols used in NADES formulations was kept constant, the above equation can also be used for quantifying excess solubility in the case of such ternary mixtures. In this case $x_{2}^{*}$ denotes the NADES mole fraction in water containing ternary solvents. The results of the application of Equation (5) are provided in Figure 7 and Figure 8 for aqueous mixtures of organic solvents and NADES, respectively. It is interesting to see that all organic solvents introduce a positive non-additivity in the whole range of concentrations except for DMSO at unimolar proportions with water. The observed small excess solubility for mixtures of this solvent with water can be attributed to the high solubility of theobromine in neat DMSO. In this case small negative value of excess solubility have been observed for a broad range of mole fractions. The strongest non-additivity is observed for 1,4-dioxane+water mixture at $x_{2}^{*\;}$= 0.3 and acetone+water at $x_{2\;}^{*}$= 0.5. Additionally, for unimolar proportions of methanol and water strong positive non-additivity is observed. Interestingly, the maximum of cooperativity between DMF and water has been noticed for $x_{2}^{*}\;$= 0.5.

Similar analysis was done for the studied aqueous NADES mixtures. As it is documented in Figure 8, very strong positive effect is observed in the whole range of solvents ratio for ionic liquids comprising glycerol or glucose and at NADES concentration exceeding $x_{2}^{*}\;$= 0.2 for sorbitol and xylitol. It is interesting to notice that in all cases the highest excess solubilities were obtained for $x_{2}^{*}$ = 0.8 composition, which is also characterized by the highest synergistic effect.

The excess values were interpreted in terms of the Redlich–Kister third order polynomial [95,96], which was also implemented in Jouyban–Acree approach [47,97]. Here, the three values of the Redlich–Kister polynomial were found by obtaining the minimum of the values of root means square deviation of experimental and computed excess solubility values using the following formula:

(6) $$\Delta^{exc}ln\left(x_{1}^{bin}\left(T\right)\right)=x_{2}\cdot (1-x_{2})\overset{2}{\underset{i=0}{\sum }}\frac{J_{i}}{T}{\left(2x_{2}-1\right)}^{i}$$

**Figure 9:**
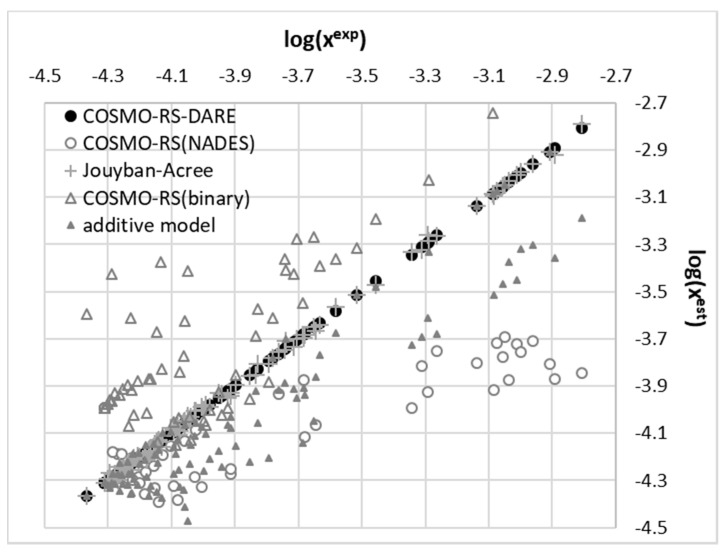
Correlation between experimental and computed values of theobromine solubility.

The application of the regression analysis leads to parameters summarized in Table 1 and the quality of fitting is graphically presented in Figure 9 for the whole set of 108 data points. The strong non-ideal mixing can be directly inferred from the distribution included in Figure 9, what is represented by distribution of values marked with gray triangles. Additionally in Figure 9, the results of a direct application of the COMOS-RS approach for solubility estimation were presented as separate distributions for binary and ternary mixtures. It is clearly visible that both series are quite poorly described based on default computations of this type with the opposite sings of estimation error. The theobromine solubility in NADES is seriously overestimated and description of binary systems suffers from underestimation. The MAPE is as high as 53% and 117% for the two series. However, there are outliers the error of which exceeds 100%. It is interesting to notice that the values of back-computed solubility of theobromine are very accurate if regression provided by Equation (6) is used for both types of systems. The values of RMSD and MAPE provided in Table 1 suggest the adequacy of using this type of fitting function for data reduction.

### 3.4. Application of COSMO-RS-DARE

**Figure 10:**
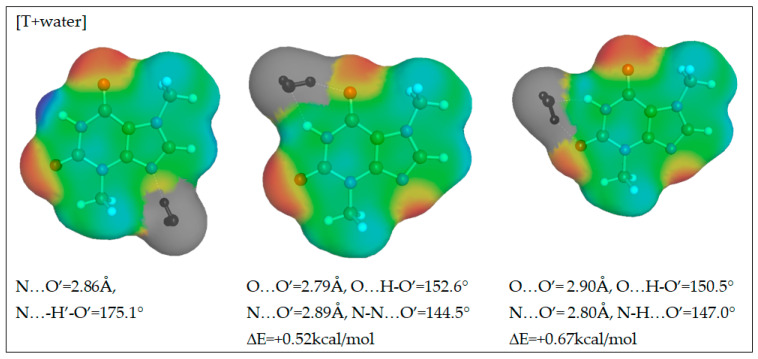
The electron density distribution of the most representative complexes of theobromine with water.

**Figure 11:**
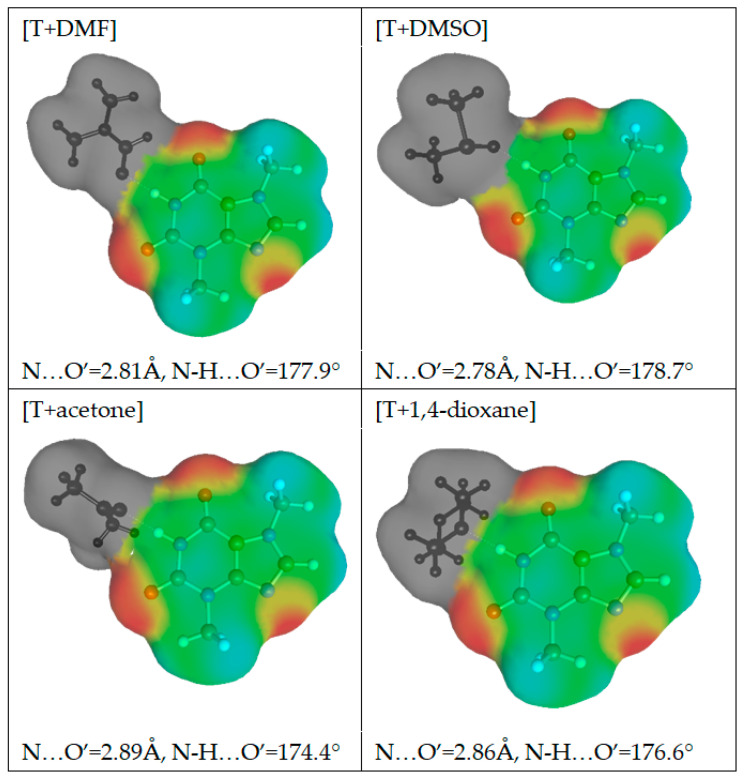
The electron distribution of the most representative complexes of theobromine with selected aprotic solvents.

**Figure 12:**
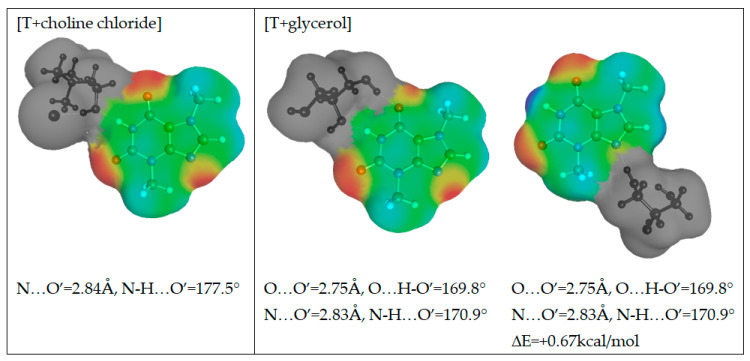
The electron distribution of the most representative complexes of theobromine with choline chloride and glycerol.

As it was mentioned in the above section, the application of a polynomial function for the approximation of solubility is very efficient and offers high accuracy of back-computed solubility values. Unfortunately, apart from proper interpolation, the deeper insight is missing what was the main motivation for application of the alternative approach termed COSMO-RS-DARE. This type of computations is an extension of the default application of COSMO-RS by redefinition of the studied systems through direct inclusion of strongly interacting molecules, which results in formation of intermolecular complexes. Additionally, in the context of the observed complex behavior of excess solubility, it is interesting to identify the contributions coming from the most dominant interactions between solution components. Theobromine can interact with solvent molecules due to its amphiprotic properties. It possess a proton donating nitrogen atom at the pyrimidine ring and a non-methylated nitrogen atom at the imidazole fragment. This allows for diverse contacts and even dimer formation. The former site will be active with such solvents as DMSO, DMF, acetone and 1,4-dioxane. Water and alcohols can be involved in interactions with theobromine utilizing both sites. To further explore this idea, direct contacts of theobromine were analyzed in terms of the energy and structure of the most probable pairs formed between solute and solvent. Hence, all of new components of the mixtures used for solubility predictions can be represented by a X + Y = XY scheme, where X and Y represent any of the solution components and XY stands for the pair involving theobromine. In the case of X = Y the theobromine dimer is obtained, otherwise hetero molecular complexes are considered. The structures of all these pairs were derived from extensive conformation analysis according to the description provided in the methodology section. Detailed information on structural parameters of structures identified as the most stable are provided in Supporting Materials in Figures S1–S4. Here, only exemplary pairs are discussed. The most interesting case is the interaction of theobromine with water. In Figure 10 the three most energetically favorable pairs are presented in the format corresponding to the “mcos” file directly used in COSMO-RS computations. As it was already mentioned, the gray part of electrostatic potential covering atoms of water is not used in the solubility computations since all are considered as pseudo-conformers of theobromine. It is interesting to notice that water can be attracted by theobromine not only by all electronegative centers but also by the acidic hydrogen atom connected to the carbon atom situated in between carbonyl groups of the six-membered ring. The most stable water–theobromine complex is formed by the acceptor from the imidazole ring, however other hydrogen bonded pairs are very close in energy. Similar type of contacts can be found for interactions of theobromine with alcohols. To the contrary, the only favorable contacts of non-protic solvent molecules such as DMSO, DMF, 1,4-dioxane or acetone are possible with the most acidic site of theobromine (N1-H). Schematic representations of electrostatic densities are provided in Figure 11. Taking into account the geometric features of the formed hydrogen bonds, it is possible to conclude that they all belong to strongly bonded complexes. Indeed, HB bonds are short and HB angles almost right, suggesting a highly linear hydrogen motive. In NADES solutions there are possible alternative structures, much richer in hydrogen boding. This is not only related to the higher number of donating centers but also to higher flexibility of polyols, resulting in larger number of potential conformations. Two exemplary cases are provided in Figure 12, demonstrating the most favorable pairs of theobromine with choline chloride and glycerol. The structure of choline chloride intermolecular pairs with theobromine is similar to the one found in the cases of non-protic solvents and by analogy it should be also regarded as very strong. Glycerol is able to utilize two hydroxyl groups for embracing both oxygen centers of Theobromine. Additionally, an alternative motive can be observed, which is only slightly energetically disfavored. This pair is stabilized by a hydrogen bond formed between the acceptor center of theobromine (N center located on imidazole ring) and donor coming from one of hydroxyl groups. Other polyols can form analogical pairs, which structural characteristics is provided in Supplementary Materials.

**Figure 13:**
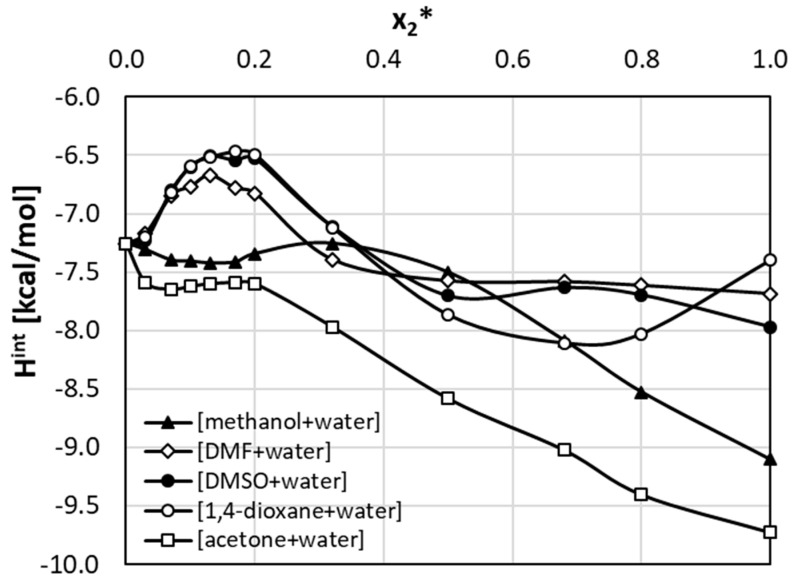
The values of interaction parameter H^int^ used in COSMO-RS-DARE computations of theobromine solubility in aqueous binary mixtures of organic solvents at room temperature.

**Figure 14:**
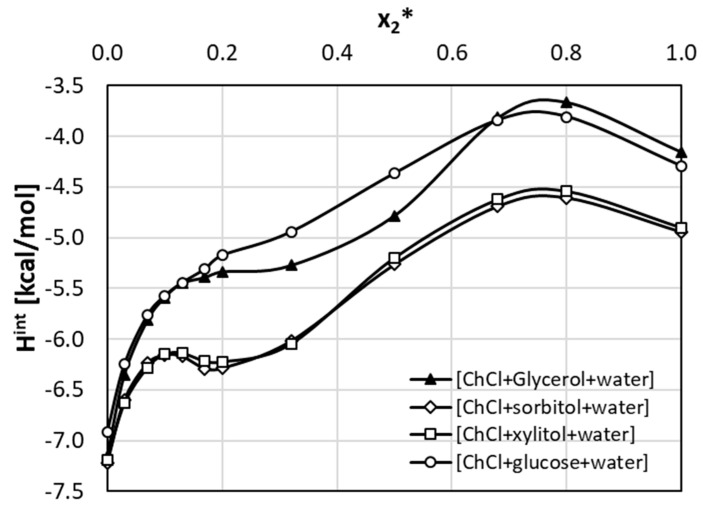
The values of interaction parameter H^int^ used in COSMO-RS-DARE computations of Theobromine solubility in aqueous ternary NADES systems at room temperature.

Augmentation of the monomeric forms with the most energetically favorable pairs involving theobromine offers a much better representation of the conformational space of analyzed systems. As it is documented in Figure 9, a serious increase of computed solubility accuracy has been achieved using COSMO-RS-DARE formalism. The quality of obtained prediction also exceeds the one resulting from parameters fitting of polynomial defined in Equation (6). This significant increase of accuracy was achieved at the cost of introducing additional interaction parameters. The values of optimized energy of interactions were provide in Figure 13 and Figure 14 for both types of dissolution media. The provided plots show a strong non-linear trend suggesting complex concentration dependency. This is not surprising in the light of discussed diversity of potential interactions of theobromine with solvent molecules.

## 4. Conclusions

Theobromine is an active pharmaceutical ingredient that is poorly soluble in many organic solvents, hence the need for alternative solvents. The performed experiments revealed that using binary mixtures of water and some organic solvents increases its solubility compared to neat solvents. Such behavior was observed in the case of 1,4-dioxane, methanol and acetone. For all of these solvents the maximum value of theobromine solubility was achieved in the case of mixture corresponding to unimolar proportions of both the organic solvent and water. The highest increase in solubility was observed for dioxane-water mixture, as it was almost four-times higher than for the neat solvent. Interestingly, no such effect was observed for DMSO and DMF, i.e., neat solvents offering the highest solubility of theobromine. Several natural deep eutectic solvents comprising choline chloride and one of seven polyols were also tested for possible theobromine solubility improvement and the obtained results confirmed their applicability for this task. The three most efficient NADES compositions, utilizing choline chloride with glycerol, sorbitol and xylitol were found to be more efficient than DMSO and all of the studied compositions turned out to be more effective than the 1:1 dioxane-water mixture. The best performing NADES, utilizing glycerol as the second constituent, offered 1.5-times higher solubility than DMSO. However, it was found that a relatively small addition of water to a NADES system results in even larger solubility of theobromine. Aqueous ternary NADES compositions in which the amount of water was equal to 0.2 molar fractions were characterized by about 20–25% higher solubility than pure eutectics, which is an important find in terms of practical application of this type of solvents. The measured solubilities were interpreted in terms of three theoretical frameworks. The first one belonging to the set of data reduction techniques proved to be very efficient in quantitative back-computations of excess solubility of Theobromine in all studied systems. This approach was augmented with two approaches originating from first principle computations. The appealing nature of such methods, which require only chemical structure does not need to be advocated. Unfortunately, the default approach utilizing the well-recognized COSMO-RS framework offered at most a qualitative description of the solubility of theobromine in studied aqueous binary and ternary NADES systems. It was suggested that one of the origins of such poor correlation between experimental solubility values and the ones computed using COSMO-RS can be attributed to inadequate system definition. The extended search for possible contacts provided evidence for the existence of many intermolecular complexes, which alter the electron density of the solute molecule. Hence, apart from monomeric structures of theobromine and solvent molecules, it is very probable that binary contacts play a non-marginal role in the total pool of interactions. Taking into account such intermolecular contacts seriously increased the accuracy of solubility computations. Unfortunately, experimental solubility data are still indispensable for proper accounting of interactions of included pseudo-conformers. However, accuracy of computed solubility is even better than the one resulting from Redlich–Kister third order polynomial fitting to the experimental data.

## Figures and Tables

**Table 1:** Collection of parameters of Jouyban–Acree equation (Equations (5) and (6)) providing the best match to experimental data for analyzed systems.

System	J0	J1	J2	RMSD·10^2^	MAPE
methanol(aq)	784.64	−111.98	−602.24	0.20	0.18%
DMF(aq)	488.43	−530.04	821.38	0.43	0.36%
DMSO(aq)	−24.07	−252.39	1030.18	0.37	0.33%
1,4-dioxane(aq)	1092.55	−917.56	176.42	0.16	0.14%
acetone(aq)	1218.77	201.83	−472.90	0.35	0.29%
[ChCl+glycerol](aq)	955.38	1056.22	320.15	0.42	0.42%
[ChCl+sorbitol](aq)	1137.84	1035.05	−822.39	0.33	0.25%
[ChCl+xylitol](aq)	1005.15	1120.46	−753.17	0.24	0.22%
[ChCl+glucose](aq)	1081.72	743.51	−185.31	0.27	0.22%

## Data Availability

Not applicable.

## Supplementary Materials

The following are available online at https://www.mdpi.com/article/10.3390/pharmaceutics13081118/s1, Table S1: (a). Concentrations of theobromine solutions and the corresponding absorbance values, together with mean absorbance and standard deviation values, used during preparation of the calibration curve. (b). Parameters of the obtained calibration curve for theobromine solubility determination. Table S2: Comparison of theobromine solubility values at 25 °C obtained in this study and the results taken from literature. Table S3: Mole fractions (10^4^) and standard deviation values (10^4^) of theobromine at 25 °C in neat organic solvents and water. Table S4: Mole fractions (10^4^) and standard deviation values (10^4^) of theobromine at 25 °C in binary solvents comprising water and an organic solvent in different proportions. Table S5: Mole fractions (10^4^) and standard deviation values (10^4^) of theobromine at 25 °C in pure natural deep eutectic solvents comprising choline chloride and the second constituent. Table S6: Mole fractions (10^4^) and standard deviation values (10^4^) of theobromine at 25 °C in mixture containing water and natural deep eutectic solvents in different proportions. Figure S1: The electron distribution of the complexes of theobromine with water and alcohols (part 1). Figure S2: The electron distribution of the complexes of theobromine with alcohols (part 2). Figure S3: The electron distribution of the complexes of theobromine with organic solvents. Figure S4: The electron distribution of the complexes of theobromine with NADES constituents.
