# Supplementary material for: Experimental and Theoretical Study on Theobromine Solubility Enhancement in Binary Aqueous Solutions and Ternary Designed Solvents
Source: Pharmaceutics. 2021 Jul 22;13(8):1118. doi: 10.3390/pharmaceutics13081118 (PMC8401494; doi:10.3390/pharmaceutics13081118)
Supplement: Supplementary file 1 [file pharmaceutics-13-01118-s001.zip › pharmaceutics-1301879-supplementary.pdf]

pharmaceutics

Supplementary Materials: Experimental and Theoretical Study on Theobromine Solubility Enhancement in Binary Aqueous Solutions and Ternary Designed Solvents

Tomasz Jeliński, Dawid Stasiak, Tomasz Kosmalski and Piotr Cysewski

Table S1a. Concentrations of Theobromine solutions and the corresponding absorbance values, together with mean absorbance and standard deviation values, used during preparation of the calibration curve.

c [mg/mL]	A_{mean}	SD
0.01600	1.057	0.021
0.01280	0.865	0.018
0.01067	0.717	0.017
0.00914	0.629	0.014
0.00800	0.525	0.017
0.00711	0.474	0.023
0.00640	0.432	0.012
0.00582	0.391	0.013
0.00533	0.354	0.015
0.00492	0.320	0.017
0.00457	0.300	0.006
0.00427	0.289	0.008

Table S1b. Parameters of the obtained calibration curve for Theobromine solubility determination.

Parameter	Value
a	66.857
b	$-1.11 \cdot 10^{-16}$
R^2	0.9987
LOD	$4.47 \cdot 10^{-4}$ mg/ml
LOQ	$1.34 \cdot 10^{-3}$ mg/ml

Table S2. Comparison of Theobromine solubility values at 25 °C obtained in this study and the results taken from literature.

Solvent	x_T [$\cdot 10^4$]		Relative Difference
	Literature	Results	
**Water** [a]	0.47	0.49	3.29%
**Methanol** [a]	0.43	0.43	−1.62%
**1-Propanol** [a]	0.21	0.20	−0.93%
**Ehtyl acetate** [a]	0.16	0.16	−2.23%
**Acetone** [a]	0.22	0.23	4.93%

[a] Zhong et al. [80].

Table S3. Mole fractions (10^4) and standard deviation values (10^4) of Theobromine at 25 °C in neat organic solvents and water.

Solvent	$x_T \cdot 10^4$	$SD \cdot 10^4$
**DMSO**	8.204	0.043
**DMF**	3.049	0.046
**1,4.-Dioxane**	1.650	0.029
**Acetone**	0.234	0.001
**Acetonitrile**	0.162	0.004
**1-Propanol**	0.204	0.002
**1-Butanol**	0.197	0.002
**1-Pentanol**	0.157	0.003
**Ethyl acetate**	0.155	0.083
**Water**	0.489	0.007
**Methanol**	0.430	0.011

Table S4. Mole fractions (10^4) and standard deviation values (10^4) of Theobromine at 25°C in binary solvents comprising water and an organic solvent in different proportions.

$x_{solvent}$	DMSO		DMF		1,4-dioxane		Methanol		Acetone	
	$x_T \cdot 10^4$	$SD \cdot 10^4$	$x_T \cdot 10^4$	$SD \cdot 10^4$	$x_T \cdot 10^4$	$SD \cdot 10^4$	$x_T \cdot 10^4$	$SD \cdot 10^4$	$x_T \cdot 10^4$	$SD \cdot 10^4$
**0.00**	0.489	0.007	0.489	0.007	0.489	0.007	0.489	0.007	0.489	0.007
**0.03**	0.581	0.046	0.664	0.005	0.604	0.003	0.513	0.004	0.498	0.013
**0.07**	0.716	0.003	0.837	0.005	0.807	0.013	0.525	0.001	0.507	0.004
**0.10**	0.816	0.008	0.905	0.007	1.002	0.014	0.556	0.005	0.546	0.012
**0.13**	0.912	0.009	1.045	0.002	1.141	0.015	0.579	0.004	0.594	0.008
**0.17**	1.016	0.010	1.116	0.006	1.398	0.021	0.620	0.003	0.673	0.016
**0.20**	1.194	0.003	1.263	0.005	1.609	0.015	0.683	0.001	0.738	0.018
**0.32**	1.465	0.011	1.480	0.003	2.064	0.017	0.842	0.003	0.874	0.020
**0.50**	1.820	0.134	1.932	0.012	2.230	0.009	0.866	0.021	0.891	0.029
**0.68**	3.506	0.175	2.321	0.059	1.968	0.013	0.714	0.004	0.734	0.012
**0.80**	5.131	0.028	2.621	0.097	1.808	0.013	0.590	0.001	0.514	0.026
**1.00**	8.204	0.043	3.049	0.046	1.650	0.029	0.430	0.011	0.234	0.009

Table S5. Mole fractions (10^4) and standard deviation values (10^4) of Theobromine at 25 °C in pure Natural Deep Eutectic Solvents comprising choline chloride and the second constituent.

NADES Constituent	$x_T \cdot 10^4$	$SD \cdot 10^4$
**Glycerol**	12.341	0.682
**Fructose**	6.589	0.723
**Glucose**	7.138	0.755
**Sorbitol**	8.940	1.200
**Xylitol**	8.343	0.938
**Saccharose**	3.938	1.011
**Maltose**	3.671	0.951

Table S6. Mole fractions (10^4) and standard deviation values (10^4) of Theobromine at 25 °C in mixture containing water and Natural Deep Eutectic Solvents in different proportions. Acronym ChCl represent Ccholine chloride.

X_{NADES}	ChCl + Glycerol		ChCl + Sorbitol		ChCl + Xylitol		ChCl+Glucose	
	$x_T \cdot 10^4$	$SD \cdot 10^4$	$x_T \cdot 10^4$	$SD \cdot 10^4$	$x_T \cdot 10^4$	$SD \cdot 10^4$	$x_T \cdot 10^4$	$SD \cdot 10^4$
**0.00**	0.489	0.007	0.489	0.007	0.489	0.007	0.489	0.007
**0.03**	0.594	0.002	0.536	0.004	0.517	0.001	0.552	0.001
**0.07**	0.651	0.002	0.576	0.002	0.544	0.005	0.630	0.010
**0.10**	0.721	0.004	0.626	0.002	0.599	0.005	0.701	0.003

**0.13**	0.830	0.003	0.698	0.002	0.659	0.002	0.806	0.004
**0.17**	0.990	0.004	0.788	0.003	0.742	0.003	0.942	0.007
**0.20**	1.218	0.005	0.945	0.004	0.871	0.003	1.217	0.037
**0.32**	2.257	0.009	2.077	0.009	1.725	0.008	2.082	0.012
**0.50**	5.111	0.021	5.459	0.023	4.867	0.023	4.542	0.045
**0.68**	12.834	0.191	9.755	0.037	8.818	0.093	8.211	0.040
**0.80**	15.594	0.170	10.960	0.033	10.026	0.053	9.216	0.085
**1.00**	12.341	0.212	8.940	0.116	8.435	0.135	7.263	0.072

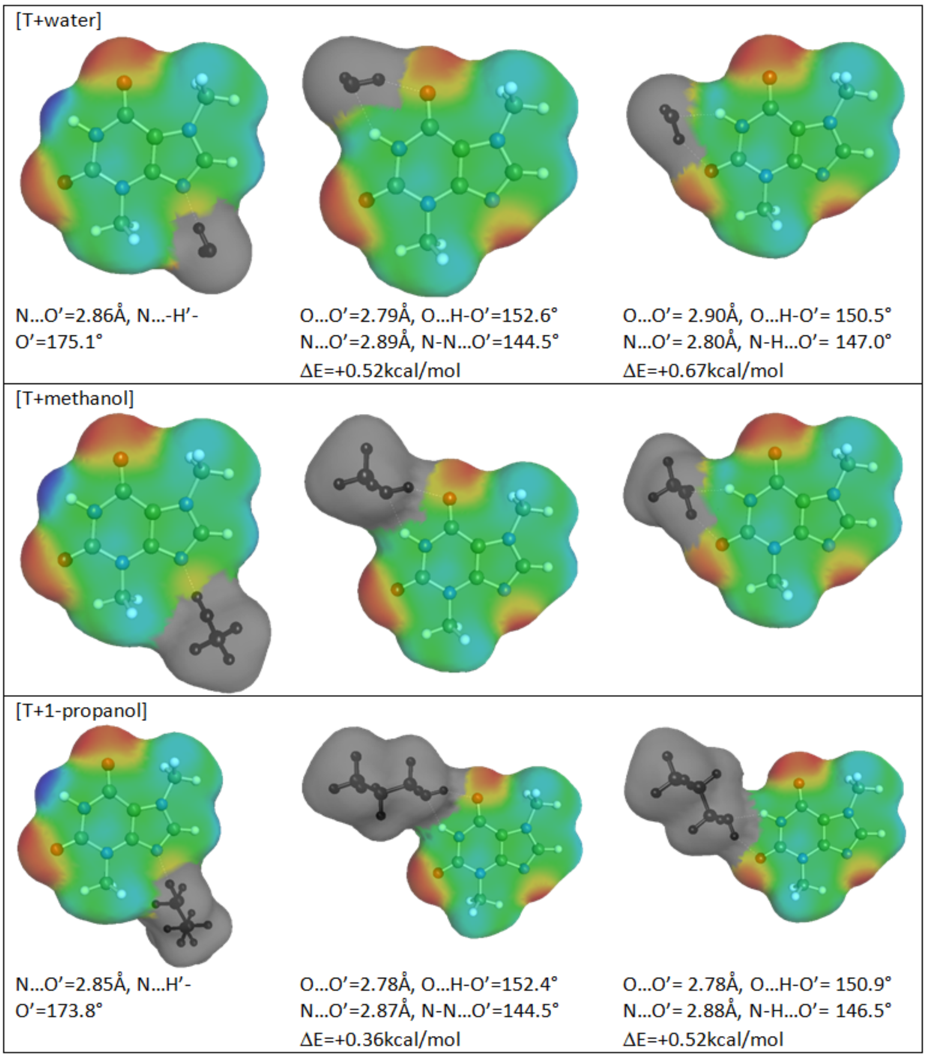

Figure S1. The electron distribution of the complexes of Theobromine with water and alcohols (part 1).

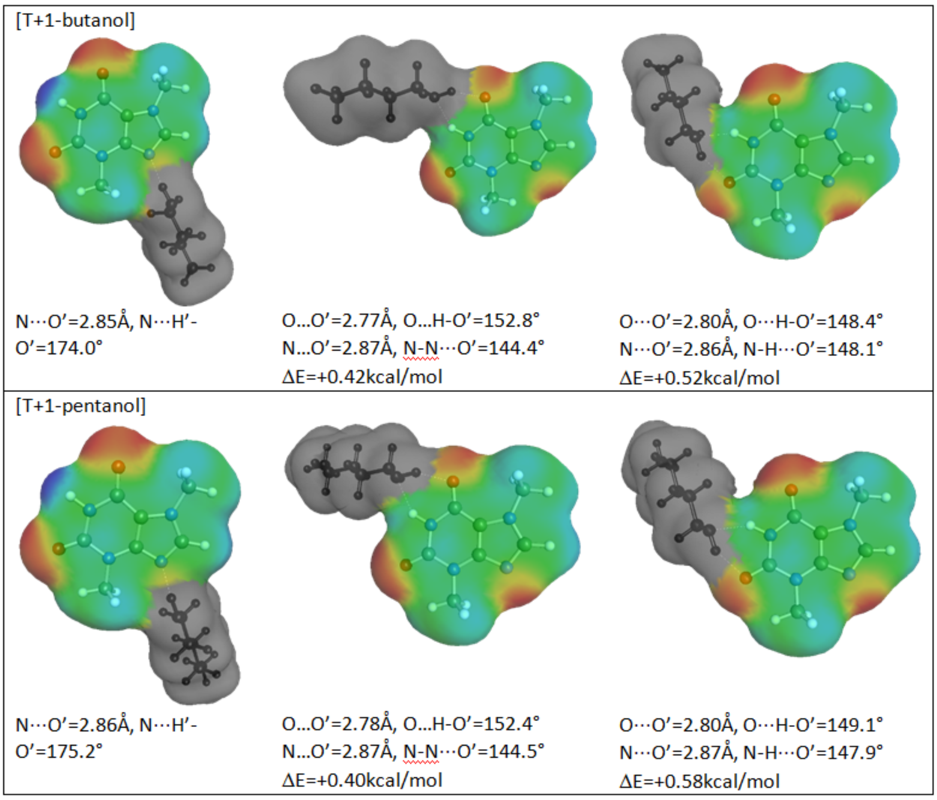

Figure S2. The electron distribution of the complexes of Theobromine with alcohols (part 2).

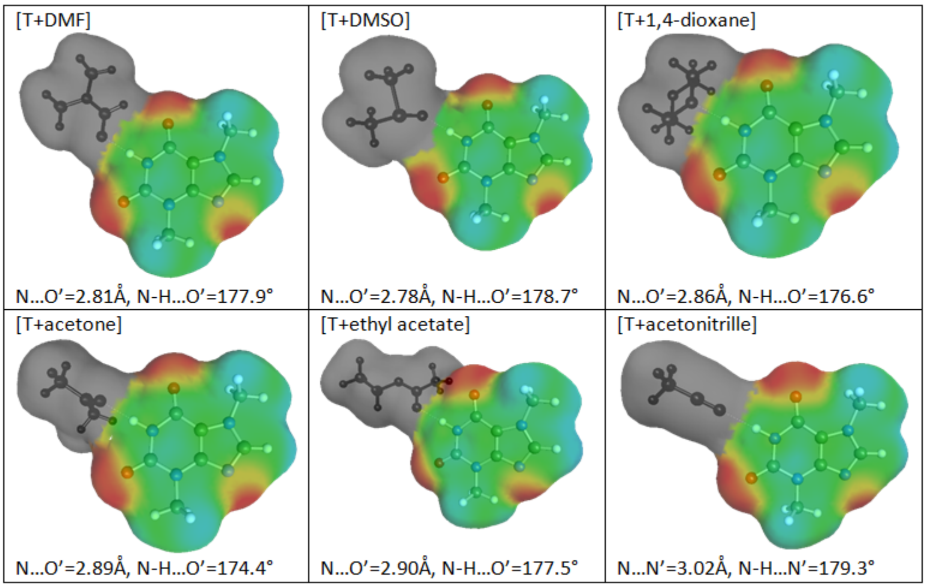

Figure S3. The electron distribution of the complexes of Theobromine with organic solvents.

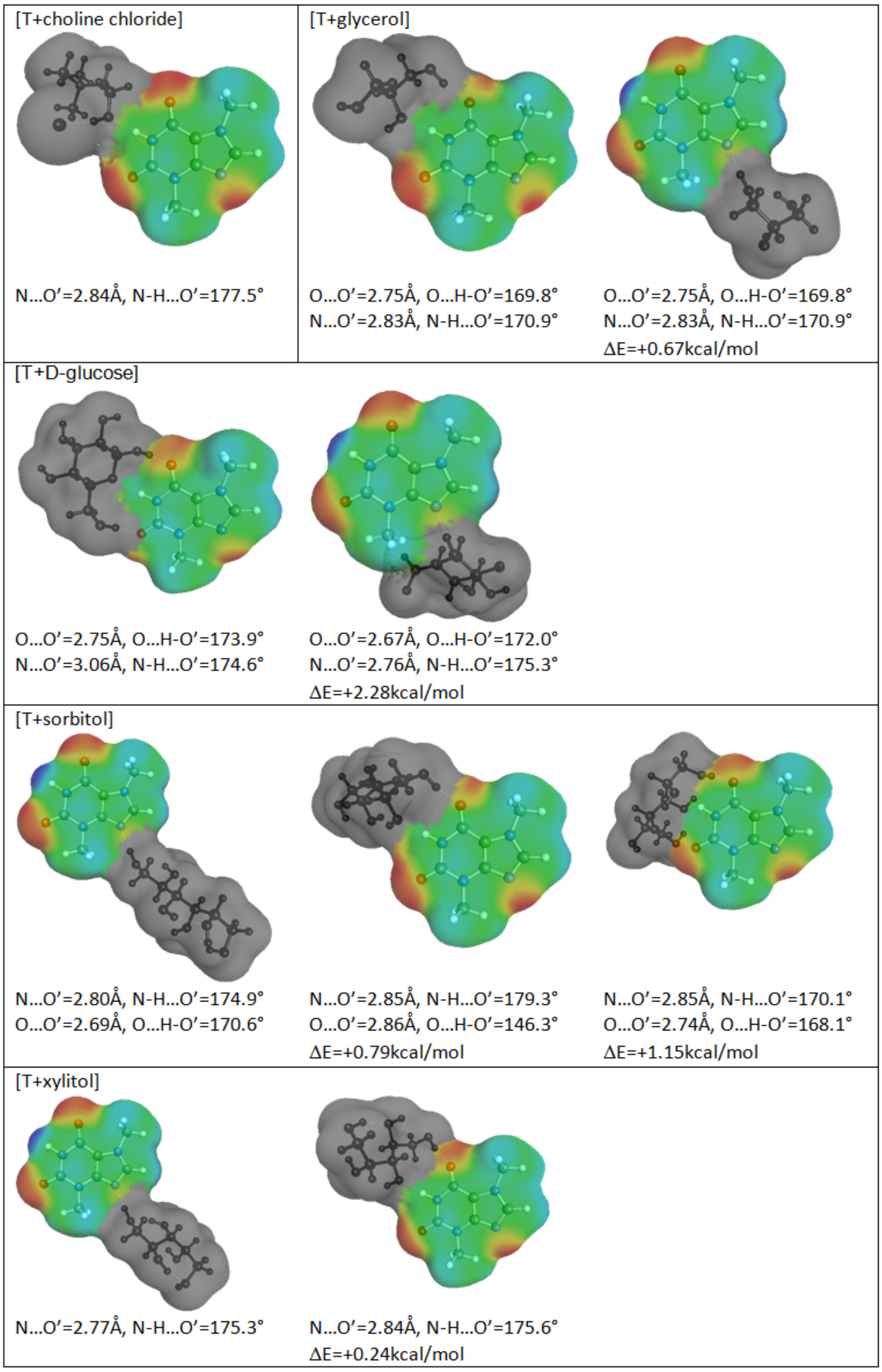

Figure S4. The electron distribution of the complexes of Theobromine with NADES constituents.
